# Supplementary material for: Changes in Inner Retina Thickness and Macular Sensitivity in Patients with Type 2 Diabetes with Moderate Diabetic Retinopathy
Source: Biomedicines. 2023 Nov 4;11(11):2972. doi: 10.3390/biomedicines11112972 (PMC10669203; doi:10.3390/biomedicines11112972)
Supplement: Supplementary file 1 [file biomedicines-11-02972-s001.zip › biomedicines-2629805-supplementary.pdf]

Supplementary Material

	RNFL OS		RNFL OT		RNFL OI		RNFL ON		RNFL IS		RNFL IT		RNFL II		RNFL IN		RNFL C global	
	Correlation coeffcient	p	Correlation coeffcient	p	Correlation coeffcient	p	Correlation coeffcient	p	Correlation coeffcient	p	Correlation coeffcient	p	Correlation coeffcient	p	Correlation coeffcient	p	Correlation coeffcient	p
Colesterol	0.041	0.770	0.002	0.987	0.074	0.592	0.105	0.452	0.231	0.093	-0.087	0.533	0.160	0.247	0.111	0.423	0.004	0.977
HDL	-0.080	0.565	-0.121	0.384	-0.122	0.380	-0.094	0.498	-0.085	0.543	**-0.362**	**0.007**	-0.246	0.073	-0.221	0.108	**-0.292**	**0.032**
LDL	0.091	0.515	-0.034	0.810	0.092	0.510	0.081	0.560	**0.285**	**0.037**	-0.100	0.471	0.145	0.297	0.118	0.397	-0.001	0.996
TG	-0.099	0.475	0.133	0.336	0.040	0.774	-0.002	0.987	-0.043	0755	0.143	0.303	0.155	0.264	0.086	0.535	0.214	0.120
FG	-0.010	0.945	-0.082	0.554	0.127	0.361	0.000	0.998	-0.027	0.848	-0.212	0.124	-0.034	0.809	-0.218	0.113	**-0.465**	**0.000**
Creatinine	0.114	0.413	0.148	0.286	-0.081	0.559	0.027	0.846	0.112	0.420	**0.280**	**0.040**	0.065	0.641	**0.271**	**0.048**	**0.452**	**0.001**
BCVA	0.082	0.554	-0.165	0.233	**-0.301**	**0.027**	-0.226	0.101	0.141	0.310	0.128	0.357	-0.157	0.257	**0.351**	**0.009**	0.252	0.066
S.E.	0.033	0813	-0.121	0.384	-0.231	0.093	-0.129	0.354	**-0.318**	**0.019**	-0.003	0.980	-0.068	0.627	0.156	0.261	0.033	0.811
A.L.	0.058	0.676	0.201	0.146	0.259	0.059	0.236	0.086	**0.416**	**0.002**	0.227	0.098	0.299	0.028	0.210	0127	**0.318**	**0.019**
IOP	-0.025	0.860	-0.190	0.170	-0.093	0.504	0.043	0.757	-0.117	0.401	-0.141	0.309	-0.035	0.801	-0.106	0.447	-0.239	0.082

Suppl. Table S1: *Correlations between clinical variables (HDL, high-density lipoprotein; LDL, low-density lipoprotein; TG, triglycerides; FR, filtrate rate; BCVA, best-corrected visual acuity; SE, spherical equivalent; AL, axial length; IOP, intraocular pressure) in DM2 patients and RNFL thickness by OCT in the nine areas of the early treatment diabetic retinopathy study (ETDRS) grid (OS, Outer Superior; OT, Outer Temporal; OI, Outer Inferior; ON, Outer Nasal; IS, Inner Superior; IT, Inner Temporal; II, Inner Inferior, IN: Inner Nasal; C, Central; and where temporal quadrants are represented left and nasal quadrants are represented right). The values that reached statistical significance ($p<0.05$) are shown in bold with a grey background.*

	GCL+ OS		GCL + OT		GCL + OI		GCL + ON		GCL + IS		GCL + IT		GCL + II		GCL + IN		GCL + C global	
	Correlation coeffcient	p	Correlation coeffcient	p	Correlation coeffcient	p	Correlation coeffcient	p	Correlation coeffcient	p	Correlation coeffcient	p	Correlation coeffcient	p	Correlation coeffcient	p	Correlation coeffcient	p
**Colesterol**	0.103	0.478	0.103	0.477	0.191	0.183	0.113	0.434	0.084	0.563	0.072	0.621	0.171	0.234	0.166	0.251	0.008	0.958
**HDL**	-0.205	0.153	0.051	0.723	-0.116	0.422	-0.026	0.860	**-0.288**	**0.043**	**-0.298**	**0.035**	-0.245	0.087	-0.109	0.405	**-0.379**	**0.007**
**LDL**	-0.114	0.421	0.056	0.699	0.049	0.737	0.128	0.376	0.067	0.641	0.070	0.630	0.077	0.597	0.360	0.275	-0.065	0.652
**TG**	**0.296**	**0.037**	-0.029	0.843	0.185	0.197	0.146	0.313	**0.307**	**0.030**	0.215	0.133	**0.296**	**0.037**	0.179	0.214	0.264	0.063
**FG**	0.144	0.319	0.097	0.505	0.181	0.208	0.205	0.153	0.028	0.844	0.003	0.984	0.118	0.415	-0.055	0.704	**-0.371**	**0.008**
**Creatinine**	-0.123	0.393	-0.014	0.921	-0.122	0.397	-0.189	0.189	-0.029	0.844	0.064	0.660	-0.078	0.591	0.120	0.407	**0.475**	**0.000**
**BCVA**	-0.242	0.090	-0.094	0.516	-0.152	0.292	**-0.226**	**-0.291**	0.041	0.374	-0.067	0.643	-0.064	0.658	-0.131	0.363	0.158	0.273
**S.E.**	-0.113	0436	-0.033	0.818	-0.115	0.427	-0.141	0.328	0.068	0.641	0.021	0.887	-0.017	0.909	-0.129	0.371	-0.026	0.857
**A.L.**	0.074	0.610	-0.135	0.349	-0.109	0.452	-0.109	0.451	-0.117	0.419	0.003	0.984	-0.139	0.337	-0.064	0.657	**0.325**	**0.021**
**IOP**	**-0.292**	**0.039**	-0.169	0.240	-0.182	0.206	-0.212	0.139	**-0.377**	**0.007**	**-0.348**	**0.0013**	-0.231	0.107	-0.334	0.018	-0.362	0.010

Suppl. Table S2: *Correlations between clinical variables (*HDL*, high-density lipoprotein;* LDL*, low-density lipoprotein;* TG*, triglycerides;* FR*, filtrate rate;* BCVA*, best-corrected visual acuity;* SE*, spherical equivalent;* AL*, axial length;* IOP*, intraocular pressure) in DM2 patients and GCL+ (GC-IPL) thickness by OCT in the nine areas of the early treatment diabetic retinopathy study (ETDRS) grid (OS, Outer Superior; OT, Outer Temporal; OI, Outer Inferior; ON, Outer Nasal; IS, Inner Superior; IT, Inner Temporal; II, Inner Inferior, IN: Inner Nasal; C, Central; and where temporal quadrants are represented left and nasal quadrants are represented right). The values that reached statistical significance (p<0.05) are shown in bold with a grey background.*

	GCL++ OS		GCL ++ OT		GCL ++ OI		GCL ++ ON		GCL ++ IS		GCL ++ IT		GCL ++ II		GCL ++ IN		GCL ++ C global	
	Correlation coeffcient	p	Correlation coeffcient	p	Correlation coeffcient	p	Correlation coeffcient	p	Correlation coeffcient	p	Correlation coeffcient	p	Correlation coeffcient	p	Correlation coeffcient	p	Correlation coeffcient	p
**Colesterol**	-0.024	0.864	0.155	0.269	0.051	0.717	-0.026	0.854	0.047	0.738	0.125	0.373	0.160	0.253	-0.006	0.966	0.056	0.691
**HDL**	--0.181	0.194	-0,036	0.799	-0.110	0.435	-0.049	0.729	-0.244	0.079	-0.250	0.070	-0.197	0.158	-0.161	0.148	**-0.383**	**0.005**
**LDL**	-0.056	0.689	0.138	0.323	0.058	0.682	-0.005	0.974	0.059	0.675	0.073	0.603	0.144	0.305	0.020	0.890	0.021	0.882
**TG**	0.081	0.564	0.027	0.845	0.041	0.771	0.037	0.790	0.150	0.285	0.197	0.157	0.167	0.233	0.073	0.605	0.255	0.063
**FG**	0.105	0.454	0.082	0.561	0.171	0.220	0.120	0.390	0.070	0.621	-0.118	0.400	0.115	0.413	-0.069	0.625	**-0.383**	**0.008**
**Creatinine**	-0.048	0.733	-0.052	0.714	-0.095	0.500	-0.106	0.451	-0.064	0.649	0.144	0.304	-0.082	0.558	0.135	0.335	**0.448**	**0.001**
**BCVA**	0.111	0.430	0.141	0.312	0.061	0.662	0.024	0.863	0.026	0.853	0.160	0.252	0.103	0.463	0.052	0.712	**-0.396**	**0.003**
**S.E.**	-0.123	0.379	-0.131	0.350	-0.125	0.374	-0.064	0.651	-0.200	0.152	-0.190	0.174	-0.160	0.254	-0.220	0.113	-0.307	0.025
**A.L.**	0.033	0.820	0.019	0.895	0.132	0.355	0.100	0.484	-0.134	0.348	-0.108	0.449	-0.088	0.540	0.052	0.719	-0.128	0.372
**IOP**	0.244	0.084	0.028	0.845	**0.377**	**0.006**	**0.283**	**0.044**	0.018	0.901	-0.035	0.805	0.131	0.360	0.188	0.186	-0.150	0.295

Suppl. Table S3: *Correlations between clinical variables (*HDL*, high-density lipoprotein;* LDL*, low-density lipoprotein;* TG*, triglycerides;* FR*, filtrate rate;* BCVA*, best-corrected visual acuity;* SE*, spherical equivalent;* AL*, axial length;* IOP*, intraocular pressure) in DM2 patients and GCL ++ (ILM - IPL/INL [GCC]) thickness by OCT in the nine areas of the early treatment diabetic retinopathy study (ETDRS) grid (OS, Outer Superior; OT, Outer Temporal; OI, Outer Inferior; ON, Outer Nasal; IS, Inner Superior; IT, Inner Temporal; II, Inner Inferior, IN: Inner Nasal; C, Central; and where temporal quadrants are represented left and nasal quadrants are represented right). The values that reached statistical significance (p<0.05) are shown in bold with a grey background.*
